# Regenerative Potential of DPSCs and Revascularization: Direct, Paracrine or Autocrine Effect?

**DOI:** 10.1007/s12015-021-10162-6

**Published:** 2021-04-07

**Authors:** Vincenzo Mattei, Stefano Martellucci, Fanny Pulcini, Francesca Santilli, Maurizio Sorice, Simona Delle Monache

**Affiliations:** 1 Biomedicine and Advanced Technologies Rieti Center, Sabina Universitas, 02100, Rieti, Italy; 2 Department of Experimental Medicine, “Sapienza” University, 00161 Rome, Italy; 3 Department of Biotechnological and Applied Clinical Sciences, University of L’Aquila, 67100 L’Aquila, Italy; 4 StemTeCh Group, Chieti, Italy

**Keywords:** Dental pulp stem cells, Regenerative potential, Stem cells differentiation, Angiogenesis, Revascularization

## Abstract

A new source of mesenchymal stem cells has recently been discovered, the so-called dental pulp derived stem cells (DPSCs) which therefore could represent potentially tools for regenerative medicine. DPSC originate from the neural crest and are physiologically involved in dentin homeostasis; moreover, they contribute to bone remodeling and differentiation into several tissues including cartilage, bone, adipose and nervous tissues. DPSCs have also been shown to influence the angiogenesis process, for example through the release of secretory factors or by differentiating into vascular and/or perivascular cells. Angiogenesis, that has a pivotal role in tissue regeneration and repair, is defined as the formation of new vessels from preexisting vessels and is mediated by mutual and reciprocal interactions between endothelial cells and perivascular cells. It is also known that co-cultures of perivascular and endothelial cells (ECs) can form a vascular network in vitro and also in vivo. Since DPSCs seem to have characteristics similar to pericytes, understanding the possible mechanism of interaction between DPSCs and ECs during neo-angiogenesis is dramatically important for the development of advanced clinical application in the field of regeneration.

**Graphical abstract:**

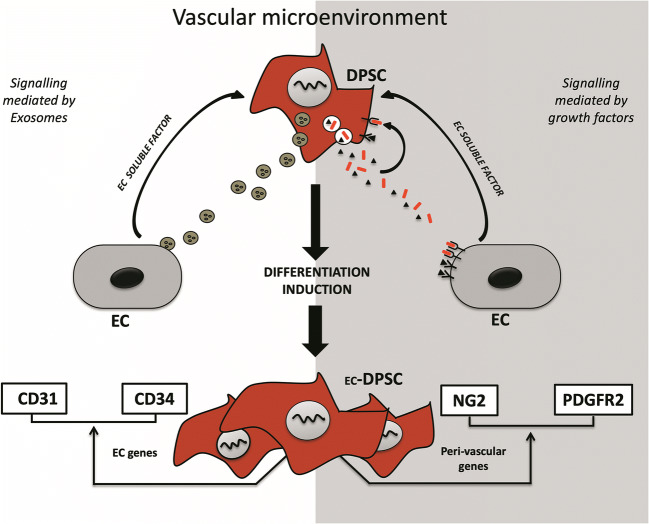

## Introduction

The stem cell is defined as a primitive, non-specialized cell with the ability to transform itself into different cytotypes through a process called “cell differentiation”. This type of cell can divide continuously by generating self-renewing or specialized cells through a process of multi-lineage differentiation.

The first evidence on the existence of stem cells is since at the beginning of the twentieth century, but the hypothesis was confirmed only in 1961 when Ernest McCulloch and James E. Till identified the self-renewing stem cells following a bone marrow transplant in mice subjected to massive doses of radiation [1].

In 1967, Altmann et al. reported evidence of adult neurogenesis and stem cell activity in the brain [2], while in 1978 Prindull discovered hematopoietic stem cells in the human umbilical cord [3]. During the last twenty years, science has made extraordinary progresses in the field of stem cells, both for the isolation and for the characterization of these cells. In 2003 Miura discovered a new source of adult stem cells in children’s milk teeth [4] and in 2006 Takahashi et al. developed a method for creating induced pluripotent stem cells (iPSCs), starting from murine fibroblast cultures [5].

To date we know that stem cells are present, in different organs and tissues within a specific physiologically limited perivascular microenvironment, called “stem niche” [6–8] where they remain quiescent and maintain their stemness characteristics [9].

Among stem cells, particularly promising are mesenchymal stem cells such as those derived from dental pulp (DPSCs). In fact, it has been shown that DPSCs possess the ability to multilineage differentiation process into both endodermal, mesodermal and ectodermal tissue [10].

## Differentiative Ability and Regenerative Potential of Dental Pulp Stem Cells(DPSCs)

The human dental pulp-derived stem cells (DPSCs) belong to mesenchymal stem cells, and as well as them, show plastic adherence and fibroblast-like morphology. It has been shown that they express specific markers for mesenchymal stem cells as CD44, CD90, CD105, CD73 and STRO-1 but don’t express hematopoietic markers like CD14 and CD19 [11].

**Fig. 1:**
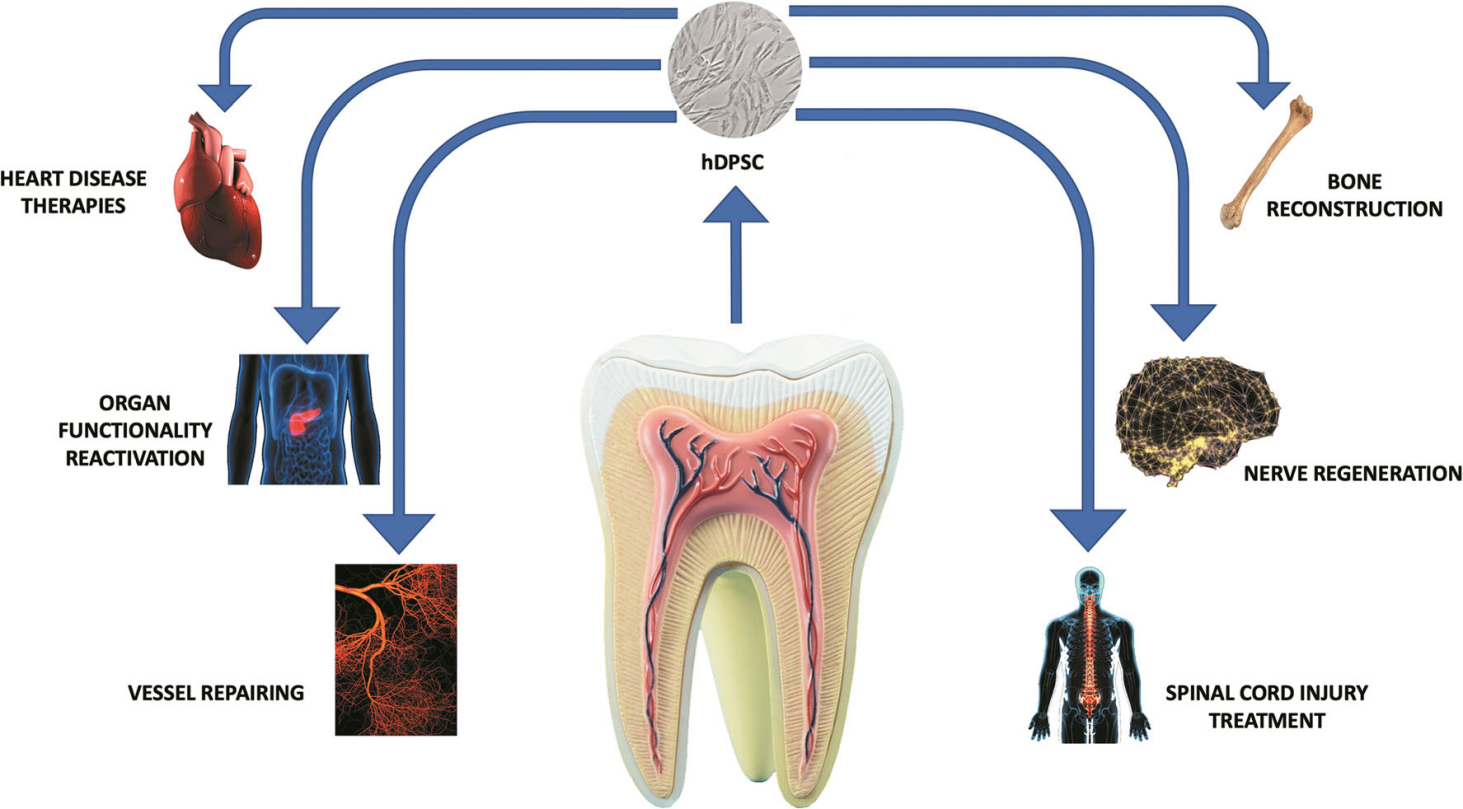
Differentiative ability and regenerative potential of Dental Pulp Stem Cells(DPSCs)

DPSCs showed in vitro the ability to differentiate into several lineages as odontoblasts, osteoblasts, chondrocytes, adipocytes, and neurons [12–14]. Besides, several scientists are studying new differentiative capacities of DPSCs. As shown in several papers and concerning to their ontogenesis, DPSCs are a highly heterogeneous population with distinct clones and expression markers as well as differences in proliferative and differentiating capacity [14]. Normally, DPSCs in the human are extracted from included third molar, in patient suffering from dysodontiasis and so that they can be considered as waste material [15]. Considering their ability, these cells could be used in regenerative medicine and in particular, given their origin, we can hypothesize their eventual future use in the regeneration of bone, cartilage, adipocytic, nervous tissues, and other tissues (Fig. 1). Indeed, several authors reported that DPSCs represent a potential new stem cell therapy for joint cartilage repair [16]. This type of cell can undergo chondrogenic differentiation and secrete the growth factors involved in tissue repair and immunomodulation. Dental pulp as an accessible source of mesenchymal cells, makes DPSCs usable instead of primary chondrocytes due to their improved cartilage proliferation and regeneration capacity [17]. One of the possible fields of application of DPSCs after chondrogenic differentiation could be the treatment of symptomatic cartilage lesions and early osteoarthritis (OA) [16]. A study conducted by Lo Monaco et al. has shown the role of DPSCs in OA, a degenerative and inflammatory joint disease with loss of cartilage [18]. The authors report the therapeutic application of DPSC in OA through immunomodulation and cartilage regeneration. Besides, non-pharmacological regeneration techniques have been developed for the regeneration of articular cartilage: in particular, it has been observed that both the use of DPSCs in vitro and the culture environment provided by particular matrices such a hydrogel, improved the process of cartilage formation [19].

Moreover, several authors confirmed by in vitro and in vivo studies that DPSCs stimulate osteogenesis and bone regeneration, showing a high proliferation rate, a good osteogenic differentiation potential, and favorable paracrine and immunomodulatory properties [19–25]. All these studies suggest that DPSCs could be used for regenerative therapies for bone-related diseases and orthopedics surgeries [25].

Also, since DPSCs express specific neural markers, such as nestin, s100-beta and GFAP or molecules as prion protein highly expressed in neuronal tissues, several authors speculated that these cells can be used as an ideal tool for neural induction and regeneration [26, 27]. In fact, DPSCs show the ability to differentiate into neuronal-like cells or dopaminergic neuron-like cells [28–30]. In addition to this ability, some works showed that DPSCs can produce and release in the medium neurotrophic factors such a brain-derived neurotrophic factor (BDNF), glial cell-derived neurotrophic factor (GDNF) and nerve growth factor (NGF). This conditional media from DPSCs can enhance the growth rate of Schwann cells and induce neurite outgrowth in vitro [29, 31]. It makes them a cellular model candidate for the study and treatment of neurodegenerative diseases, such as Alzheimer’s, Parkinson’s, and Huntington’s disease [32, 33].

Indeed, in addition to the differentiated capacities already known, recent studies have shown the differentiation of DPSCs into hepatocytes and insulin secreting beta cells [34].

### Others Differentiative and Regenerative Ability

In the field of tissue regeneration, an important achievement could be the use of MSCs (autologous or eterologous) to promote revascularization. Since DPSCs, as MSCs, possess the ability to multilineage differentiation process into both endodermal, mesodermal, and ectodermal tissue, they are considered a promising source of stem cells for regenerative therapies of several diseases [35]. Several studies demonstrated DPSCs ability to promote angiogenesis [36]. This capacity could depend on a direct differentiation of DPSCs on endothelial cells or to paracrine effects.

Moreover, DPSCs have been thoroughly studied in regenerative medicine and tissue engineering as autologous stem cells therapies and have shown amazing therapeutic abilities in oro-facial, neurologic, corneal, cardiovascular, hepatic, diabetic, renal, muscular dystrophy, and auto-immune conditions, in both animal and human models, and most recently in human clinical trials [37]. In the past ten years, several efforts were made to improve techniques for the vascularization of tissue graft. Since the regenerative capacity is directly linked to the presence of stem cells progenitors, which are capable of proliferation and differentiation, the most explored therapies to improve post-ischemic or post-infarction neovascularization have been cell-based therapies [38]. For example, several authors demonstrated in patients with peripheral artery diseases or myocardial ischemia, that autologous implantation of bone-marrow cells or endothelial progenitors cells (EPCs) could be safe and effective for the achievement of therapeutic angiogenesis, given the ability of these cells to supply the role of endothelial progenitor cells and/or to stimulate secretion of angiogenic factors or cytokines [39–41]. Furthermore, it has been shown that human mesenchymal stem cells (hMSCs) can repair ischemic tissue and restore tissue function via the formation and stabilization of new vessels and immunomodulation [42]. Besides, Tanaka et al. reported that quality controlled culture of CD34^+^ isolated cells, including the endothelial and hematopoietic progenitor-enriched fraction of EPCs, improved angiogenesis and vasculogenesis in refractory diabetic wounds [42, 43].

## DPSCs and Revascularization

The study of angiogenesis, the process of new vessel formation from pre-existing vessels, is fundamentally important when it comes to regenerative medicine, and the understanding of the cellular and molecular mechanisms underlying blood vessel regeneration is necessary to define effective and feasible therapeutic strategies. The mechanisms of regulation of the angiogenesis, vasculogenesis, and arteriogenesis processes represent a key point for the repair and regeneration of several tissues even of naturally avascular tissues such as cartilage. In this last one, the inhibition of blood vessel formation has been shown to promote the spontaneous chondrogenic differentiation of the progenitors [44]. In light of the above consideration, it is understandable that in order to develop new strategies promoting vascularization also by engineered constructs, it is necessary to study the interactions that endothelial cells can establish with other cells in every single phase of the process. In particular, it is known that DPSCs, which recently have been identified as a potential promise in the field of tissue regeneration, interact with endothelial cells which in turn regulate each other their functions.

By the way, it is well understood that to form functional vessels, participation but also interactions among a major number of different cell types such as endothelial cells, endothelial progenitors cells, macrophages, pericytes, etc. are essential [45]. Also, Rafii and Lyden, a few years ago, in a review focused on angiogenesis, have assumed that the angiogenic factors released following vascular trauma or hypoxic situations recruited a subset of pro-angiogenic hematopoietic cells, including hematopoietic stem cells (HSCs) and hematopoiet-ic progenitor cells (HPCs). Corecruitment of HSCs and HPCs together with EPCs seemed to represent the trigger that started and supported the neo-angiogenesis pro-cess [46].

Confirming this observation, Lasala et al. had found that a combination of MSCs, that are involved in smooth muscle and pericyte production during blood vessel formation and EPCs, was more effective than the single cell type in supporting the formation of a mature and stable blood vessel [47, 48]. This study, for the first time in the United States, demonstrated the feasibility, safety, and efficacy of stem cell combination therapy in patients with limb ischemia [49].

However, the regenerative potential of pericytes was already well known. For example, transplanted pericytes had been shown to increase muscle fibers number in mice with dystrophy or after muscle injury [50]. Pericytes were also implicated in wound healing in a full-thickness skin wound representing a putative source of human MSCs for use in dermal tissue engineering [51]. To clarify the origin and overlapping function of MSCs with pericytes, numerous studies have been conducted. Caplan for the first time suggested that not all pericytes are MSCs but assumed that vice-versa all MSCs are pericytes [52]. More recently, Caplan assumed that MSCs arise from perivascular cells, pericytes, in response to injury or inflammation of blood vessels. These pericytes/MSCs are present in all the vessels of our body, and in response to changes in the microenvironment, they secrete specific factors that serve the various important reparative functions into the lesion site. It has previously shown that DPSCs have pericyte-like topography and function expressing typical perycite markers. In particular, DPSCs significantly upregulate NG2, and partially alpha-smooth muscle actin [53] whereas they don’t express endothelial markers such as CD31 or von Willebrand Factor (vWF). Other authors demonstrated that the ability of DPSCs to differentiate into a perivascular lineage when co-cultured with  Human umbilical vein endothelial cells  (HUVECs) [54]. Therefore, DPSCs display phenotypes consistent with perivascular cell populations and support the process of vasculogenesis and angiogenesis not only secreting pro angiogenesis factors such as Vascular-Endothelial Growth Factor  (VEGF) but also differentiating into both endotheliocytes and pericytes [36, 55, 56].

## DPSCs Affect Angiogenesis/Regeneration Processes in Several Phases

As known sprouting angiogenesis is initiated in poorly perfused tissue when the oxygen sensing mechanism detects a low level of hypoxia. Most types of cells in this vascular niche respond to a hypoxic environment secreting VEGF, the master regulator of vascular growth. Indeed, VEGF starts the complex cascade of cellular and molecular events leading to the orderly assembly of new endothelial structures, their association with mural cells/pericytes, forming in such way fully functional vascular networks [57]. Under the role played by parenchymal cells in the vascular microenvironment, it has been shown that DPSCs, as well as the MSCs, are conditioned by the interaction with the microenvironment [58, 59]. This interaction governs proliferation potential, migration and homing, differentiation and inflammatory response of DPSCs that act as a modulator in maintaining inflammatory balance in various systemic diseases and contributing to tissue repair and regeneration [60].

### Regulation of VEGF Production

It is known that DPSCs express a plethora of proangiogenic factors and induce angiogenesis directly or by paracrine secretion [58]. Among them,VEGF is a primary angiogenic growth factor and results over-expressed by DPSCs in several works. In particular, as demonstrated by Dissanayaka et al. DPSCs seem able to triggering angiogenesis secreting VEGF that is rapidly utilized by the HUVECs to activate signaling for migration and vascular structure formation [61]. Similarly, Lee et al. demonstrated that a VEGF neutralizing antibody hindered the tubular formation of HUVECs incubated with DPSCs conditional media [62]. Also, Nagaraja et al., demonstrated VEGF release by dental pulp registering an increase of this factor at different time intervals. Moreover, VEGF expressed by dental pulp can facilitate chemotaxis, cell growth, and cell differentiation in an autocrine fashion [63].

### Enzymatic Degradation and Facilitation of EC Migration

Besides the VEGF secretion, also the secretion of other factors involved in several phases of the angiogenesis process, such as proliferation or stabilization, has been reported. For example in a study conducted by Hylkens et al., the authors demonstrated that DPSC expressed beta Fibroblast Growth Factor (bFGF), matrix metalloproteinases, endostatin thrombospondin-1 and insulin-like growth factor-binding protein-3 [64]. Some studies demonstrate the proliferative and contraction/remodeling capabilities of DPSCs within 3D type I collagen gels in vitro. They express matrix metalloproteinases and have the ability to degrade biomaterial scaffolds and to regulate cellular functions in 3D environments contributing to the process of vascular remodeling [65].

The influence of DPSCs on proliferation, migration and tube formation through the secretion of aforementioned factors has been reported both in vitro and in vivo*,* for example, in a rat model of myocardial infarction under basal conditions and after injury or hypoxia [35, 64, 66–68]. In these studies, self-renewal ability and immunomodulatory capacity of DPSCs vs Bone-marrowed (BM)-MSCs have been compared demonstrating their higher pro-angiogenic potential. Moreover, DPSCs as BM-MSCs result able to secrete VEGF but also insulin-like growth factor 1 and 2 (IGF-1/IGF-2), stem cell factor (SCF) and granulocyte stimulation factor (G-CSF) [69, 70].

Thus, an important conclusion extrapolated from several types of research, is that VEGF alone may not be able to promote tissue regeneration and that the combination with other growth factors has a stronger and more comprehensive role in promoting tissue regeneration. Among pro-angiogenic factors, also the role of transforming growth factor-beta (TGF-β) has been considered to be associated with tissue remodeling. In fact, in a study conducted by Muppala et al., they demonstrated that TGF-β, probably in association with other factors, upregulates the production of TSP-4, a proangiogenic extracellular matrix protein in cultured endothelial cells (EC), thereby promoting angiogenesis [71].

Another important aspect of the angiogenic cascade is the endothelial migration process. In particular, Hylkens et al. demonstrated that DPSCs express multiple factors which are known to affect migration, such as Angiopoietin-1 (ANGPT1), EDN1, IGFBP3, uPA and VEGF [64, 72]. Following 24 h of incubation, in a chemotactic assay, DPSCs resulted significantly able to increase endothelial transmigration.

### Sprouting, Pericytes Recruitment and Vessel Survival and Stabilization

A final phase of the angiogenesis process is represented by sprouting and vessel stabilization. ANGPT-1, a critical player in vessel maturation binds to Tie-2 receptors on endothelial cells stimulating vessel sprouting and pericyte recruitment [73]. It has been demonstrated that DPSCs secrete Ang-1 determining a vessel stabilization even though is currently not clear the mechanism of interaction between DPSCs, perycites and endothelial cells [74].

For example, a significant vascularization mediated by co-cultures of DPSCs and HUVECs, in which probably DPSCs act as endothelial or peri-endothelial cells, has been observed, confirming their role in a later stage of the angiogenesis process [61]. Moreover, some authors suggest that DPSCs can support angiogenesis associating with vessels resembling pericyte-like cells and stabilizing them [53, 55] . These studies provide indications about a new mechanism(s) of DPSC angiogenic induction and their function as pericytes, crucial aspects for DPSC use in tissue regeneration. Intriguingly Janebodin et al. suggest a mechanism of action in which DPSCs, by phosphorylation of MEK1/ERK signaling and activation of the downstream transcriptional factor ERG, leads to expression of VE-cadherin, which is required for anastomosis of DPSC-derived blood vessels [53]. In conclusion, these results unveiled a signaling pathway that enables the generation of functional blood vessels upon vasculogenic stimulus of DPSCs. [56]

## DPSCs Regenerative Mechanism: Paracrine or Direct Differentiation

As described in this review, depending on the microenvironment, the MSCs/pericytes have several key roles. Firstly, they inspect the damage, isolate foreign components, stabilize the injured tissue, provide antibiotics and then start the sequence of events to regenerate the damaged tissue [75]. In a nutshell they act as a therapeutic agent starting the process of regeneration and checking its completion step by step. All these results support the idea that at least a subset of pericytes/MSCs in our body have regenerative potential and differentiate into other lineages to restore or replenish tissue [76]. Recently, scientific community came to think that regeneration capacity of a tissue could depend not only on the interactions and participation of multiples cell types, but above all from the interactions among all these elements that can occur even from the release of growth factors, inflammatory cytokines, vesicles or exosomes and/or others molecules. For example, Spiller et al., starting from the point that the natural inflammatory response is a major regulator of vascularization processes, investigate ability of macrophage to stimulate secretion of growth factors and cytokines. They demonstrate, in a murine subcutaneous implantation model, even though in contrast with the previous paradigm, that the secretion of VEGF, the major regulator of angiogenesis, is promoted mainly by primary human macrophages (M1). Nonetheless, they show that M2 macrophages secrete the highest levels of Platelet-derived Growth Factor-BB (PDGF-BB), an important factor acting as a chemoattractant and in stabilizing pericytes, and also promote anastomosis of sprouting endothelial cells in vitro and secrete other proteases involved in vascular remodeling [77].

Besides, CD133^+^ BM-MSCs seem to activate several factors through a paracrine mechanism to help tissue regeneration, modifying endometrial behaviour through an immunomodulatory milieu that precedes proliferation and angiogenic processes. Insight into these processes could bring us one step closer to a non-invasive treatment for Asherman’s syndrome and endometrial atrophy (AS/EA) patients [78].

Therefore, in addition to control wound healing and regeneration by immunomodulatory regulation mediated by cell-to-cell interaction or by indirect secretory signaling, all these factors can mediate the generation of host cells of new tissue through paracrine signaling which induce differentiated cells in the tissue to direct regeneration and angiogenesis [79].

Paracrine signaling can also include exosomes or extracellular vescicles (EVs) that recently have emerged to be as novel cell-free vector in target tissue [80]. For example the use of exosomes and existing clinical material (collagen tape) for dental pulp tissue regeneration have been tested, in animal models, as a regenerative endodontic treatment [81].

Interestingly, the importance of exosomes has been also investigated for neuro-regeneration. Exosomes derived from human exfoliated deciduous teeth (SHEDs) has been considered for the treatment of neurodegenerative disorders such as Parkinson as new potential therapeutic tool [82].

Nevertheless, it remains unclear the extent to which stem cell-based therapies are able to induce regeneration in various organs and tissues and in what way the degree to which clinical outcomes could be improved. In general, it has been established that the regenerative potential of exogenous and endogenous cells can be mediated by vesicles, immunomodulatory regulation or direct differentiation in a tissue-specific way and that these cells have per se the potential to modulate the function of blood vessels in different stages as follows; inflammatory phase and/or hypoxic condition, homing and recruitment phase, enzymatic degradation of capillary basement membrane, activation and proliferative phase, directed migration of endothelial cells, tube maturation and pericyte stabilization [79]. On the other hand, a complex and dynamic inter-cellular communication between ECs, DPSCs and microenvironment generated by secretory signaling may lead to DPSCs differentiation to support vascular network formation (graphical abstract).

### Paracrine Mechanism of DPSCs

DPSCs express several different proangiogenic factors, which are able to induce angiogenesis in a paracrine way, by expression of paracrine angiogenic factors, such as VEGF, bFGF and PDGF under basal conditions, after injury or hypoxia. It was shown that these cells can promote in vitro endothelial cell migration and tube formation and in vivo angiogenesis and tissue regeneration [72]. Indeed, Hilkens et al.

smartly demonstrated that DPSCs significantly promoted endothelial cell migration [64]. DPSCs also had a pronounced effect on endothelial tubulogenesis, as revealed by an in vitro Matrigel™ assay. In addition, a sustained pro-angiogenic effect of DPSCs was revealed in an in vivo setting. These data indicate a pro-angiogenic influence of DPSCs in vitro, suggesting that these cells can promote the vascularization of regenerate dental tissues. Paracrine effects of DPSCs also involve different molecules such as cytokines, which play an important role in promoting and controlling the differentiation and activation of peripheral cells. Cytokines commonly expressed in DPSCs are involved in various mechanisms, including dental development, neurogenesis, inflammatory responses, regeneration of the dentin–pulp complex and vascularization [62, 83]. Indeed, in addition to Tumor necrosis factor alfa (TNFα), which is a typical proinflammatory cytokine, various chemotactic cytokines were found to be present in DPSCs. Interestingly, previous studies have shown that proinflammatory cytokines, including TNFα, also induce the odontogenic differentiation of DPSCs. In addition, cytokines associated with odontoblast differentiation (NT-3, BMP-4, TGF-β1, and TGF-β3) were strongly expressed in DPSCs. NT-3 is a neurotrophic cytokine and in human pulp cell culture it is able to increase mRNA level of dentin sialo phosphoprotein, which is a typical phenotype marker for odontoblasts. BMP-4 is also related to dentin formation, since it increases the expression of alpha-I collagen mRNA that is essential for dentin formation. TGF-β1, and TGF-β3 are multifunctional cytokines belonging to the TGF superfamily that have been shown to signal the induction of odontoblast like cell differentiation and upregulation of their matrix secretion in the human dentin–pulp complex. In particular, TGF-β1 is known to be an important promoter of both DPSC migration and dentin formation, thus contributing to the regeneration of the dentin–pulp complex. Also, cell proliferation-related cytokines may be expressed in DPSCs, In particular, Insulin-like growth factor-binding protein 6 (IGFBP-6) serves as a carrier protein of IGF-1 and helps to prolong the half-life of circulating IGFs in all tissues. Generally, IGF-1 and IGF- binding proteins are found in the undifferentiated mesenchymal component of the dental pulp, which is expressed in the apical complex during root formation. Thus, DPSCs play a role in providing a microenvironment for regeneration of the dentin–pulp complex by expression of odontoblast-differentiation-related cytokines. An additional mechanism by which DPSCs might exert effect on cell differentiation is represented by extracellular vescicles (EVs), which might possess proangiogenic paracrine effects [58]. These EVs comprise exosomes, microvesicles and apoptotic bodies, which can be differentiated based on size and origin. They contain proteins, DNA and miRNA, and play an important role in cell-cell communication. Even thoughthe potential role of EVs derived from DPSCs in angiogenesis and cell differentiation is still controversial, several evidence demonstrate their potential role in different physiopathological conditions, including myocardial infarction, neurological disorders, traumatic brain injury, stroke, hind limb ischemia and wound healing, induced by their proangiogenic properties. Several proteins, such as Angpt-1 and TIMP-1, which play a role in all steps of the angiogenic process, i.e., ECM degradation, endothelial cell proliferation, migration, were detected in EVs from DPSCs [58]. They were able to activate both the Wnt4/b-catenin signaling and the NF-kB pathway. Thus, Xian et al. showed a positive effect of DPSC-derived exosomes on endothelial cell proliferation and tube formation [84], which was further confirmed and extended by Gonzalez-King et al. [85]. Since MVs can be stored stably for several months, off-the-shelf products and cell-associated complications can be avoided, their potential therapeutic application in regenerative medicine could be promising.

### Direct Differentiation of DPSCs

The use of DPSCs in pre-clinical studies has increased considerably in the last decade as a promising alternative source of mesenchymal stem cells because of their relatively easy isolation [85, 86]. During this time the scientists have formulated several hypotheses about the biological mechanism of action of these cells in tissue repair and regeneration also referring to what is described in the literature for the MSCs [85].

As for MSCs, the goal of in vivo applications of DPSCs is mainly based on their ability to differentiate into various cell types and in particular in endothelial cells. Nevertheless, also for the DPSCs, the current paradigm cannot exclude the alternative and maybe coexisting mechanism called the paracrine effect, in which DPSCs can secrete biologically active molecules to promote angiogenesis and regeneration.

One of the first impressive demonstrations of the involvement of DPSCs in the generation of neo-vasculature was obtained by Luzuriaga et al. in 2019. These researchers explored the possibility to differentiate genetically unmodified DPSCs in endothelial cells using a commercial serum-free neural stem cell (NSC) growth medium with the presence of heparin, EGF/FGF, and B27. They found that DPSCs grew in this medium and differentiated in endothelium and showed the ability, when grafted into the brain of immuno-compromised nude mice, to be integrated into murine vasculature towards endothelial/pericyte lineage [87].

Maybe DPSCs more than Adipose derived stem cells (ADSCs) are able to differentiate in endothelial cells and to promote regeneration as demonstrated by Jin and colleagues. In particular, they demonstrated a significant increase of VEGF and PECAM-1 (CD31) angiogenesis-related gene expression in DPSCs rather than in ADSCs. Also, the amount of VEGF determined by ELISA by DPSC groups was significantly higher than that of the ADSC group. [88]

Investigating new and viable approaches for enhancing pulp regeneration, Zou and colleagues found that DPSCs treated with Semaphorin-4D (Sema4D) (2 μg/ml) seemed to acquire endothelial phenotype and characteristics showing an increase of EC specific angiogenic genes and protein expression profile and exhibiting similar endothelial vessel formation ability of HUVECs [36].

Therefore, as described, DPSCs can be induced to differentiate into vasculogenic endothelial (VE) cells even though the process that results in tube formation of DPSCs-derived vessels remains partially unexplained.

In recent works, we demonstrated that DPSCs subjected to specific culture conditions changed their phenotype differentiating in a population over-expressing pericyte markers and that in vitro could stabilize tube networks. The capacity of DPSCs to interact and bind endothelial cells seemed to be modulated by expression of specific proteins such as N-Cadherin, an important regulator of a heterotypic binding between the cell types [55].

This relevant aspect has been taken up and studied by Sasaki and colleagues transplanting VE-cadherin-silenced and controls primary human DPSCs into subcutaneous space of immunodeficient mice [56].

In particular, they observed that VE-cadherin silenced cells generated fewer functional blood vessels (anastomosed with the host vasculature). Moreover, DPSCs stably transduced with a VE-cadherin reporter, demonstrated that VEGF was able to induce VE-cadherin expression during sprouting of DPSCs but not in quiescent DPSCs. These results can unveil a mechanism that enables the vasculogenic differentiation of DPSCs and subsequently allows the generation of functional blood vessels [56].

## Therapeutic Use of DPSCs in Angiogenesis-Related Diseases

Angiogenesis is the process of forming new blood vessels from pre-existing vessels and is involved in the regulation of both physiological (such as embryonic development and wound repair) (phisiological angiogenesis) and pathological processes (pathological angiogenesis). In cases of pathology, this process can be caused either by a low angiogenic activity as occurs in cardiovascular diseases (CVDs), ischemic heart disease (IHD), myocardial infarction (MI), stroke, atherosclerosis, coronary artery disease (CAD) or by a high angiogenic activity, as occurs in oncological pathologies or chronic inflammations [89]. There is currently great interest in the search for new therapies to be applied for the treatment of CVDs because they are the leading cause of disability and death in the World [90].

Among the various therapeutic approaches in regenerative medicine, the use of stem cells for tissue engineering is beginning to take hold [91]. In particular, cell therapy (CT), including that involving the use of DPSCs, appears to be a promising innovation for the regrowth of damaged tissues since DPSCs obtained from dental pulp are an attractive, non-invasive, easy to isolate and cultivate tool [92, 93] and can be an interesting strategy for the treatment of CVDs, in particular ischemic stroke (IS) [94] and various diseases, such as spinal cord injury, Parkinson’s disease, Alzheimer’s disease, muscular dystrophy, diabetes, liver disease, eye disease, immune disease and oral disease [86, 95].

Stem cell therapy is used for various degenerative diseases; in particular Song M, et al., studying Ischemia, demonstrated both in vivo in a rat stroke model and in vitro in an ischemia model, that DPSCs isolated from tooth pulp may be a better source in CT for IS than hBM-MSC [96]. Iohora et al. demonstrated the therapeutic action of DPSCs which induce increased blood flow and high density of capillary formation in a rat model with hind limb ischemia [97]. In another study, Nito et al., demonstrated in a rodent ischemia model that DPSC transplant improves functional recovery of brain damage following acute cerebral ischemia [98]. Since there is currently no chemical or biological therapy that has been shown to actively improve neurological recovery during the chronic post-stroke phase, CT with DPSCs appears to be an excellent option for improving post-stroke disability. Preclinical evidence indicates an improvement in neurobehavioral function after autologous DPSC therapy in adult stroke survivors with chronic disability. These data will be evaluated in a phase I clinical trial to determine the maximum tolerable dose of autologous DPSCs therapy;to define that DPSC therapy at the maximum tolerable dose is safe and feasible in chronic stroke and to estimate the efficacy parameters required to design  a future Phase II/III clinical trial [99].

Early phase studies of CT in human stroke subjects indicate a functional benefit and safety of cell therapy in treatment strategies for patients with stroke-related disabilities [99]. Also, transplanted DPSCs exhibited neuroprotective effect on brain ischemia rats, by reducing the infarct volume and enhancing the neurological function recovery after cerebral ischemic injury. Moreover, DPSCs provide a new platform for the treatment of ischemic stroke with the most attractive potential [100, 101]. The data suggest that transplanted DPSCs can survive, proliferate, migrate and localize around ischemic areas and there are several preclinical studies in which DPSCs exert a neuroprotective effect resulting in improved functional outcome and reduced infraction volumes in rodents stroke models [96, 102]. Moreover, Gandia et al., concluded that DPSCs are able to repair the infarcted myocardium with a visible increase in the number of vessels and reduction in the size of the infarct, probably due to their ability to secrete proangiogenic and antiapoptotic factors [35].

These findings indicate that dental pulp-derived cells would be useful for cell-based regenerative medicine for various diseases. However, many problems must be resolved before using this kind of cells in the clinical field. Among them, the cell’s microenvironment and their delivery methods,which affect the regeneration process, are variables that cannot be still neglected [15, 103, 104].

## Declarations.

### **Availability of data and material** (data transparency)

Not applicable for that section.
